# pH and Nitrate Drive Bacterial Diversity in Oil Reservoirs at a Localized Geographic Scale

**DOI:** 10.3390/microorganisms11010151

**Published:** 2023-01-06

**Authors:** Ying Xu, Jianwei Wang, Qingjie Liu, Qun Zhang, Jiazhong Wu, Minghui Zhou, Yong Nie, Xiao-Lei Wu

**Affiliations:** 1 State Key Laboratory of Enhanced Oil Recovery, PetroChina Research Institute of Petroleum Exploration & Development, Beijing 100083, China; 2 College of Engineering, Peking University, Beijing 100871, China; 3 Institute of Ocean Research, Peking University, Beijing 100871, China; 4 Institute of Ecology, Peking University, Beijing 100871, China

**Keywords:** microbial community, oil reservoir, assembly, microbial diversity

## Abstract

Oil reservoirs are one of the most important deep subsurface biospheres. They are inhabited by diverse microorganisms including bacteria and archaea with diverse metabolic activities. Although recent studies have investigated the microbial communities in oil reservoirs at large geographic scales, it is still not clear how the microbial communities assemble, as the variation in the environment may be confounded with geographic distance. In this work, the microbial communities in oil reservoirs from the same oil field were identified at a localized geographic scale. We found that although the injected water contained diverse exogenous microorganisms, this had little effect on the microbial composition of the produced water. The Neutral Community Model analysis showed that both bacterial and archaeal communities are dispersal limited even at a localized scale. Further analysis showed that both pH and nitrate concentrations drive the assembly of bacterial communities, of which nitrate negatively correlated with bacterial alpha diversity and pH differences positively correlated with the dissimilarity of bacterial communities. In contrast, the physiochemical parameters had little effect on archaeal communities at the localized scale. Our results suggest that the assembly of microbial communities in oil reservoirs is scale- and taxonomy-dependent. Our work provides a comprehensive analysis of microbial communities in oil reservoirs at a localized geographic scale, which improves the understanding of the assembly of the microbial communities in oil reservoirs.

## 1. Introduction

Oil reservoirs are typical deep subsurface biospheres, which exhibit extreme environmental conditions, such as high salinity and high temperature [1,2]. Diverse microorganisms have been found in oil reservoirs using culture-dependent and culture-independent approaches, such as sulfate-reducing microorganisms, nitrate-reducing microorganisms, fermenters, acetogens, and methanogens [3,4,5,6]. They affect the quality of crude oil, mediate methanogenesis, and drive the biogeochemical process in the deep subsurface [1,7].

Despite significant efforts to reduce our dependence on fossil fuels for energy, crude oil remains one of the most important resources for industry and energy. Numerous technologies for enhanced oil recovery have been developed, of which microbial enhanced oil recovery (MEOR) is one of the most economic, sustainable, and efficient approaches [8]. Despite its unique advantages, this technique has not been widely applied, as conditions in oil reservoirs such as high temperature, high pressure, anoxic conditions, and high salinity are extremely harsh for microbial survival. Understanding microbial metabolism and the assembly of microbial communities in oil reservoirs is therefore essential. An adequate understanding of the relationship between microbial community structure and oil reservoir conditions is critical to the success of enhanced oil recovery.

Although the oil reservoir is a slow, diffusion-driven deep biosphere environment, it has been anthropogenically perturbed and has been transformed into an advection-driven system during oil exploration and production [2]. Along with the water injection, new electron acceptors, donors, and exogenous microbes are introduced to the previously static environment, which reassembles the microbial community in the oil reservoir. Numerous studies have investigated microbial diversities, metabolic potential, and the assembly of microbial communities in water-flooded oil reservoirs [6,9,10,11,12]. Microorganisms utilize hydrocarbons and complex refractory organic matter as carbon and energy sources before water flooding [13]. As the external water is injected, aerobic hydrocarbon degradation, nitrate reduction, H_2_S, sulfide, and sulfur oxidation become more active [2,12]. Moreover, the microbial composition of oil reservoirs also changes with anthropogenic perturbations. The dominant microorganisms in oil reservoirs shift from slow-growing anaerobes such as members of the Thermotogales and Clostridiales to fast-growing opportunists such as members of the Deferribacteres, Delta-, Epsilon- and Gammaproteobacteria [2]. A ‘core’ microbiota consisting of three bacterial genera, including *Arcobacter*, *Pseudomonas*, and *Acinetobacter*, and eight archaeal genera, most of which are methanogens, was found to be present in the production water samples from eight water-flooded oil reservoirs throughout northern China [14]. In contrast, Delta/epsilon-proteobacteria (e.g., sulfate-reducing species) together with methanogens (mostly *Methanococcus* species) are the most abundant groups in high-temperature oil reservoirs located in the Norwegian Sea that have not been subjected to any sea-water injection [13,15]. It is notable that the higher the in situ temperatures of the oil reservoirs are, the less the effects of microorganisms in the injected waters on microbial community compositions in the produced waters [16]. As the temperature increases, the microbial communities in water-flooded wells become more similar to those in geographically distant oil reservoirs and non-flooded wells [15,16,17,18]. These findings suggest that in situ physiochemical conditions, including those altered by water injection, reshape the microbial composition of water-filled oil reservoirs.

The assembly mechanisms of microbial communities are essential for understanding the ecological functions of microorganisms in biogeochemical processes in ecosystems [19]. It is believed that both deterministic and stochastic processes work together simultaneously in determining the community assembly [20,21,22]. The former is generally referred to as niche-based mechanisms, including environmental filtering and various biological interactions. The latter includes random birth–death events, probabilistic dispersal, and ecological drift [23]. The geographic variability can also affect the assembly of microbial communities, which may coincide with the variability of environmental sources, leading to spurious correlations between microbial and environmental variables [24,25]. Continent-scale biogeography has been studied in oil reservoir ecosystems, and the effects of environmental variables on the microbial communities at the continental scales have been investigated. It has been reported that both the bacterial and the archaeal communities in oil reservoirs show clear distance-decay patterns at a large geographic scale [26], which suggested that the microorganisms in oil reservoirs tend to be locally adapted or are dispersal limited [27]. The variation in the environment is likely to be partially confounded with geographic distance, making it difficult to determine the roles of physiochemical parameters in the assembly of microbial communities in oil reservoirs. Although recent research revealed that physiochemical parameters such as temperature and pH affect the diversity of microbial communities in oil reservoirs at a large geographic scale [14,26], it is unclear how the local environmental factors drive the assembly of oil reservoir microbial communities.

In this study, to investigate the influence of physiochemical parameters on the assembly of microbial communities, independent of variations in geographic distance, we collected produced water samples from 26 production wells and three injected water samples from the same flooded oil field. All wells have similar natural histories and geologic conditions but are distinguished by different flooding durations and anthropogenic perturbations. They have similar temperatures and depths, but different chemical properties. High-throughput sequencing of 16S rRNA genes was used to analyze both bacterial and archaeal communities. Correlation analysis between physiochemical data and microbial diversity has improved our understanding of how environmental variability shapes microbial communities in oil reservoirs at local geographic scales.

## 2. Materials and Methods

### 2.1. Sample Collection and Geochemical Analysis

All samples in this study were collected in July 2021 from production and injection wells in the Changqing field, a high-temperature, low-permeability oil field in northwest China. This oil field has been exploited over 10 years by long-term water flooding. The shallow groundwater is the major source of the injection water with an in situ temperature of about 20 °C. Three samples of the injection wells (i.e., Z1, Z3, and Z4) and 26 samples of the production wells were collected (Table 1 and Table S1), using the sampling valve on the wellhead. The bottles were filled with samples to exclude air. All bottles were sealed with sterile screw caps and then transported to the laboratory within 48 h below 5 °C after sampling for further analysis.

For the geochemical analysis, cell-free water samples were obtained by filtration through 0.22-μm cellulose ester membrane filters. Total Dissolved Solids (TDS) were measured using gravimetric method [28]. The concentrations of cations and anions in the injected and produced waters were analyzed using an ion chromatograph (SHINE CIC-D160) with an SH-AC-3 column (for cation analysis) and an SH-CC-9 column (for anion analysis).

### 2.2. DNA Extraction and 16S rRNA Gene Sequencing

The oil and water phases of the produced water was separated by gravitational precipitation. Then, approximately 500 mL of the water phase of each reservoir production sample was collected for DNA extraction. Microbial cells in the water phase were collected by filtration through 0.22-μm sterilized cellulose ester membrane filters. Genomic DNA was extracted and purified from the collected cells using the FastDNA^®^ Spin Kit for Soil (MP Biomedicals, Cleveland, OH, USA). The concentration and purity of DNA were determined by NanoDrop2000 UV–vis spectrophotometer (Thermo Scientific, Wilmington, NC, USA), and DNA quality was checked by 1% agarose gel electrophoresis.

The hypervariable region V3-V4 of the bacterial 16S rRNA gene was amplified using the conserved primers primer pairs 338F (5′-ACTCCTACGGGAGGCAGCAG-3′) and 806R (5′-GGACTACHVGGGTWTCTAAT-3′). Primers 524F10extF (5′-TGYCAGCCGCCGCGGTAA-3′) and Arch958RmodR(5′-YCCGGCGTTGAVTCCAATT-3′) were used to amplify hypervariable region V4-V5 of the archaea 16S rRNA gene [29]. PCR amplification of 16S rRNA gene was carried out in 20-μL reaction mixtures, which contained 4 μL 5 × TransStart FastPfu buffer, 250 μM dNTPs, 0.2 μM of each, TransStart 0.4 μL FastPfu DNA Polymerase (TransGen Biotech Co., Ltd., Beijing, China), 0.2 μL BSA, 10 ng template DNA, and ddH_2_O up to 20 μL. The PCR reaction for amplifying bacterial 16S rRNA gene was conducted using the following program: 3 min of denaturation at 95 °C, 22 cycles of 30 s at 95 °C, 30 s for annealing at 55 °C, and 45 s for elongation at 72 °C, and a final extension at 72 °C for 10 min. For amplifying archaeal 16S rRNA gene, the reaction cycle was after an initial denaturation at 3 min of denaturation at 95 °C, 31 cycles of 30 s at 95 °C, 30 s for annealing at 55 °C, and 45 s for elongation at 72 °C, and a final extension at 72 °C for 10 min. The resulting PCR products were extracted from a 2% agarose gel and further purified using the AxyPrep DNA Gel Extraction Kit (Axygen Biosciences, Union City, CA, USA) and quantified using QuantiFluor™-ST (Promega, Madison, WI, USA) according to the manufacturer’s protocol. The purified products were pooled in equimolar and paired-end sequenced (2 × 250) on an Illumina NovaSeq platform according to the standard protocols (Majorbio Bio-Pharm Technology Co., Ltd., Shanghai, China).

### 2.3. 16S rRNA Gene Sequence Processing and Statistical Analysis

The raw reads obtained from sequencing were quality-filtered using Trimmomatic [30]. Specifically, reads with quality scores lower than 20, or sequence lengths shorter than 100 bp, or containing N were discarded. The clean reads were analyzed using the Quantitative Insights into Microbial Ecology 2 (QIIME 2) pipeline (v2019.7) [31]. Barcodes and primers were trimmed using the Cutadapt plugin [32]. Sequences were denoised and clustered into amplicon sequence variants (ASVs) using the DADA2 plugin [33]. Taxonomy assignment of the ASVs was performed using the Silva classifier (Silva-138) [34]. Afterwards, samples were rarefied to an even depth of 50,000 sequences per sample for bacteria and 30,000 sequences per sample for archaea, alpha diversity and beta diversity were calculated using the “qiime diversity” function implemented in QIIME2. The unconstrained non-metric multidimensional scaling (NMDS) analysis, Mantel test, Spearman correlation analysis, and hierarchical clustering were performed in R (v3.6.2), with the “vegan” and “pheatmap” packages. The Sloan Neutral Community Model for Prokaryotes was employed in R as described previously [35]. Results were visualized in R (v3.6.2) using the “ggplot2” package.

## 3. Results

### 3.1. Physicochemical Characteristics of the Produced Water Samples

Recent biogeography research of microbial communities in oil reservoirs has demonstrated that the assembly of oil reservoir microbial communities is influenced by both physicochemical conditions and geographical locations [14,26]. Because of the dispersal limitation, although the oil reservoir microbial communities showed high heterogeneities at a large geographic level, such as the continental scale, it is not clear how niche selection drives the microbial communities at a localized scale (e.g., within the oil field). In this study, a total of 29 samples were collected from three injection wells and 26 adjacent production wells in the Changqing oil field, a high-temperature, low-permeability oil field in northwest China. The geographic distance between two adjacent wells is less than one kilometer. To characterize the natural habitat of the microbial community in each production well, we measured the geochemical conditions of each sample, which are listed in Table 1. The depth of the sampling wells ranged from 1505 to 1807 m and the temperature ranged from 53 to 62 °C. The most produced water samples had faintly acid pH values ranging from 6.30 to 7.20. High variations were found in the concentrations NO_3_^−^ (0–237.2 mg/L) and SO_4_^2−^ (1.83–767.5 mg/L), which were the potential electron acceptors in the anaerobic hydrocarbon degradation.

### 3.2. Microbial Compositions of the Produced Water Samples

**Figure 1:**
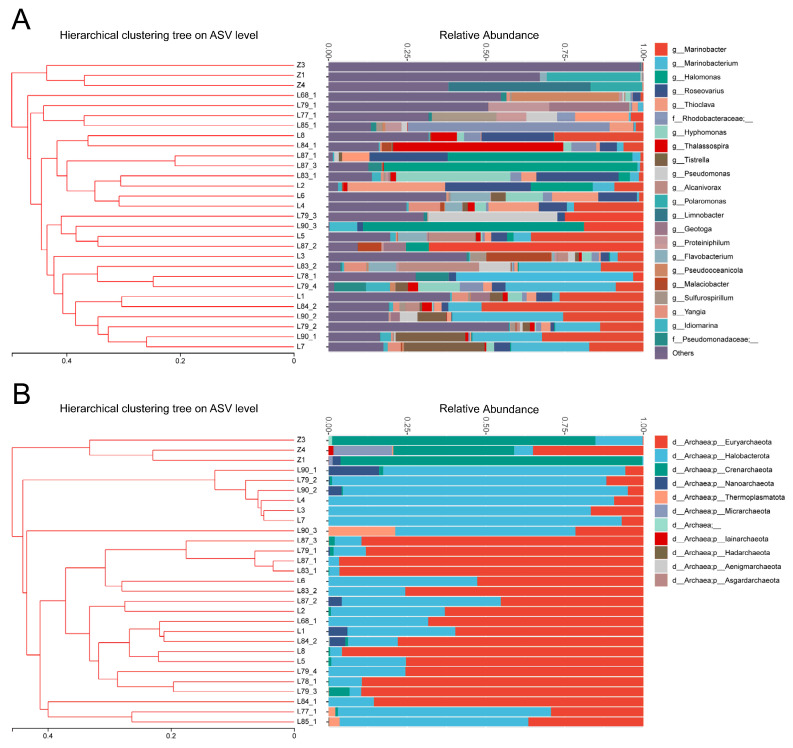
Microbial composition of the injected and produced water samples. (**A**) bacterial composition at the genus level. The taxa with average relative abundances of less than 1% were grouped as “Others”. (**B**) archaeal composition at the phylum level.

A total of 11,168,180 bacterial 16S rRNA gene sequences and 5,494,843 archaeal 16S rRNA gene sequences were obtained from all samples. A total of 2472 and 555 amplicon sequence variants (ASVs) were obtained for bacterial and archaeal communities, respectively. To investigate whether niche selection drives the microbial communities in the oil reservoirs, we compared the microbial composition of injected water samples and produced water samples. The injected water communities and produced water communities only shared 146 bacterial ASVs and 22 archaeal ASVs, which only accounted for 9.3% and 5.0% of total bacterial and archaeal ASVs in the produced water samples, respectively (Figure S1). The hierarchical clustering based on the Weighted UniFrac distances of both bacterial and archaeal communities clearly showed that all samples formed two distinct clusters as a function of the sample (Figure 1). The injected water samples clustered together and all the produced water samples formed a separate cluster. Moreover, the taxonomic analysis showed that the microbial communities in the injected water samples and produced water samples were distinguished by their dominant taxa. Although the bacterial communities of both injected water samples and produced water samples were dominated by Proteobacteria (Figure S2), they showed distinct bacterial compositions at the genus level. *Polaromonas* (average 15.3% of the total frequencies), *Limnobacter* (15.1%), *Hydrogenophaga* (7.1%), *Novosphingobium* (8.1%), and *Caulobacter* (5.9%) were the dominant genera in the injected water. In contrast, *Marinobacter* (16.4%), *Marinobacterium* (9.8%), *Halomonas* (9.4%), and *Roseovarius* (6.2%) dominated the bacterial communities of the produced water at the genus level. For archaea, the injected water samples were dominated by Crenachaeota and Euryarchaeota, but the produced water samples were dominated by Euryarchaeota and Halobacterota at the phylum level (Figure 1B). The results indicated that microorganisms in the injected water had a weak effect on the microbial composition in the produced water. Because the dispersal of microorganisms in water-flooded oil reservoirs is primarily mediated by the injected water, this result suggested that niche selection might be the key factor driving microbial communities in oil reservoirs. It is also worth noting that the effect of microorganisms in the injected water on the microbe composition in the produced water may be environment dependent. For example, because the higher the in situ temperature of a reservoir, the fewer microorganisms in the injected water are adapted in the oil reservoir, the effect of microorganisms in the injected water on the microbial composition of the produced water is weaker in the high-temperature reservoirs [16].

### 3.3. Assembly Mechanisms of Oil Reservoir Communities at a Local Geographic Scale

**Figure 2:**
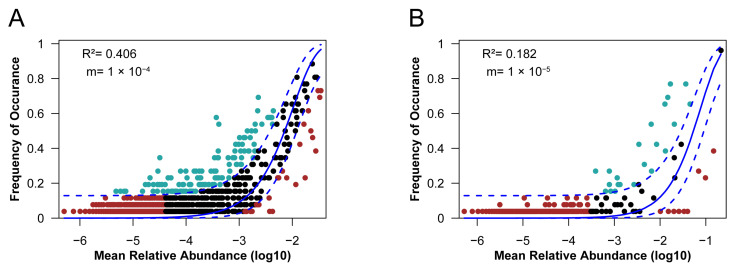
Fits of the neutral model to the produced water sample. (**A**) fit of the neutral model to the bacterial community. (**B**) fit of the neutral model to the archaeal community. Each point represents a different ASV. The solid line represents the predicted occurrence frequency of ASVs, and the dashed line represents the 95% confidence interval around the model prediction. Green points, ASVs over-represented; red points; ASVs under-represented; black points, ASVs neutrally-distributed.

To assess the neutral processes (e.g., migration) to the assembly of oil reservoir microbial communities, we employed the Slogan Neutral Community Model (NCM) [35]. We compared the observed community composition and distribution across the sample at the ASV level with that predicted by the Slogan neutral model (Figure 2). The fit of the neutral model (as indicated by R^2^) was 0.406 for the bacterial community and 0.182 for the archaeal community. The estimated migration rates were 1 × 10^−4^ and 1 × 10^−5^ for the bacterial community and archaeal community, respectively, which were extremely low compared with other surface environments [36,37,38]. The results suggested that high dispersal limitation of microorganisms occurred in oil reservoirs even at the localized scale, which was consistent with the fact that although injected water might bring exogenous microorganisms into the deep subsurface reservoirs, they had little effect on the microbial composition (Figure 1).

### 3.4. The Concentration of Nitrate Determines the Diversity of the Communities

**Figure 3:**
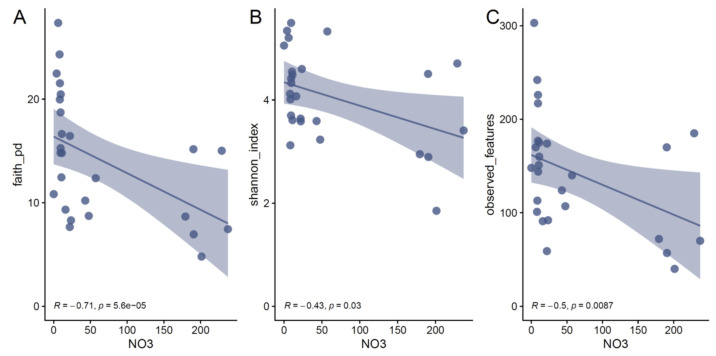
Nitrate concentration is correlated with bacterial alpha diversity in oil reservoirs at a localized geographic scale. (**A**) the correlation between the Faith’s PD index and the nitrate concentration. (**B**) the correlation between the Shannon index and nitrate concentration. (**C**) the correlation between the richness and nitrate concentration. Points represent the alpha diversity of each sample; lines represent the fit lines between nitrate concentration and alpha diversity.

Because the neutral processes (e.g., migration) had little effect on the assembly of oil reservoir microbial communities, we speculated that physiochemical parameters might determine the microbial diversity and community assembly. Correlations of individual physicochemical parameters with alpha diversity showed that the concentration of NO_3_^−^ is a major driver of variation in bacterial alpha diversity (Figure 3 and Figure S3). The Shannon–wiener index (Spearman’s coefficient: *r_s_* = −0.43, *p* = 0.03), faith PD index (Spearman’s coefficient: *r_s_* = −0.71, *p* = 5.6 × 10^−5^), and observed features (Spearman’s coefficient: *r_s_* = −0.5, *p* = 0.0087) all decreased as the NO_3_^−^ concentrations increased. Note that the evenness of the bacterial communities (i.e., Pielou index) was not correlated with the physiochemical parameters and the results indicated that high concentrations of NO_3_^−^ decreased the bacterial richness. Similar results were also found in oil reservoirs with nitrate injection [39] and nitrate-treated pipelines [40]. In contrast, no significant correlation between archaeal alpha diversity and physiochemical parameters was found (Figure S4).

### 3.5. pH Drives the Microbial Compositions of the Communities

To reveal how the physiochemical parameters determine the microbial composition in the oil reservoirs, we investigated the correlation between physiochemical parameters and the beta diversity throughout the samples. We found that beta diversity (i.e., weighted UniFrac distance) of bacterial communities in the produced water significantly correlated with pH (Mantel test: r = 0.2876, *p* = 0.005) (Table 2).

**Figure 4:**
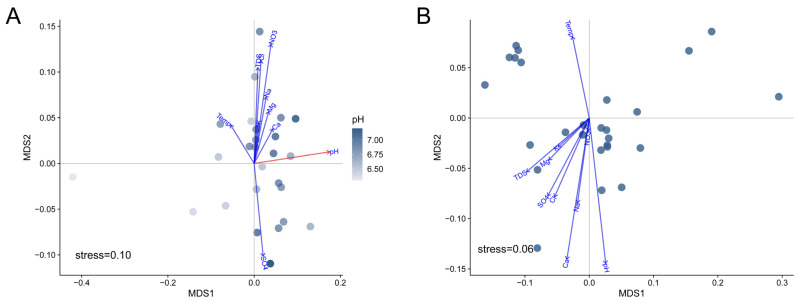
Non-metric multidimensional scaling (NMDS) ordination based on the weighted UniFrac distances of bacterial communities (**A**) and archaeal communities (**B**).

**Figure 5:**
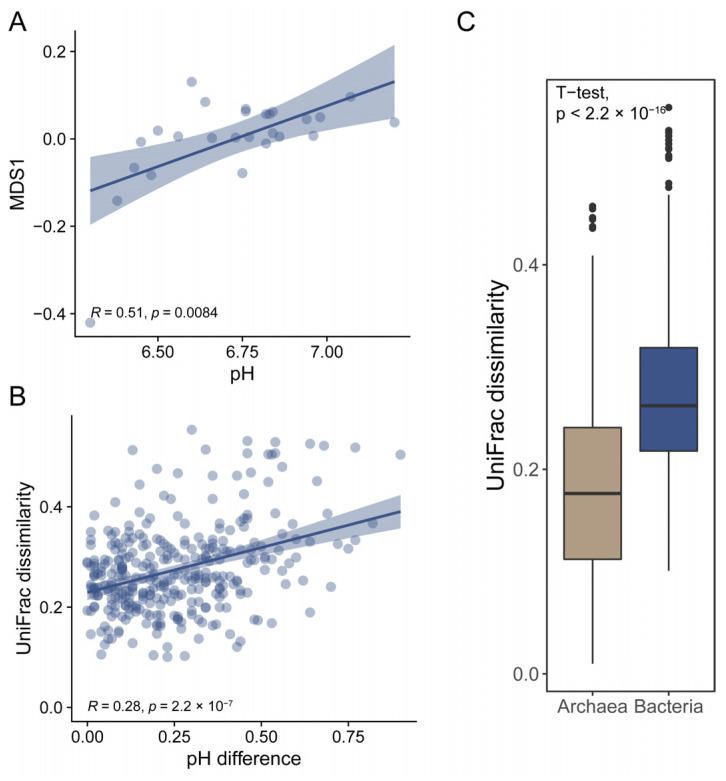
pH drives the bacterial composition in oil reservoirs at a localized geographic scale. (**A**) the relationship between the microbial profiles and pH values. Points represent the MDS1 values of each sample; line represents the fit line between pH and MDS1 values. (**B**) the relationship between beta diversity and pH difference. Points represent the UniFrac dissimilarities between each sample; line represents the fit line between pH and the UniFrac dissimilarities. (**C**) comparison of archaeal and bacterial beta diversity within the produced water samples.

Similar to the results for the correlation between archaeal alpha diversity and physiochemical parameters, the beta diversity of archaeal communities was not correlated with physiochemical parameters either. We fitted the physiochemical parameters to unconstrained non-metric multidimensional scaling (NMDS) ordination (Figure S5). The result showed that pH significantly correlated with the bacterial compositions in the produced water samples (r = 0.344, *p* = 0.01) (Figure 4A). To confirm whether pH drives bacterial communities, we compared beta diversity across samples as a function of pH. We found that nMDS 1 positively correlated with pH values for bacteria (Spearman’s coefficient: *r_s_* = 0.51, *p* = 0.0084) (Figure 5A). Weighted UniFrac distances also showed that the bacterial dissimilarities between samples were significantly correlated with the increase in pH values (Figure 5B and Figure S6). The results confirmed that pH drove the bacteria compositions of produced water samples at a localized geographic scale.

In contrast, the physicochemical parameters had a weak effect on archaeal compositions (Figure 4B and Figures S7 and S8). These results suggested that the archaeal communities were homogenized in oil reservoirs at a localized geographic scale. We compared the beta diversity within bacterial communities and archaeal communities, respectively. We found that the weighted UniFrac dissimilarities within bacterial communities were significantly higher than those within archaeal communities (Figure 5C), suggesting higher homogeneities throughout archaeal communities in oil reservoirs at a localized geographic scale. The homogeneities of archaeal communities were also found at a larger scale [14,26]. Zhao et al. found that the archaeal compositions are conserved in water-flooded oil reservoirs throughout China, which showed more core archaeal taxa [14]. Yun et al. found that although both bacterial and archaeal communities displayed clear distance-decay patterns, the beta diversity within archaeal communities was lower than that of bacterial communities [26]. These findings suggested that the archaeal community is more homogeneous in oil reservoirs at a large scale. Moreover, a stronger correlation between archaeal communities and physiochemical parameters than bacterial communities was also found at the continental scale, which might be explained by the high heterogeneity of bacteria communities at the large scale [14]. In this work, we found that at the local geographic scale the bacteria communities were significantly correlated with pH. The results suggested that the assembly of microbial communities in oil reservoirs is scale- and taxonomy-dependent, i.e., archaeal compositions could be predicted at a large geographic scale, while bacterial compositions could be predicted at the local scale by physiochemical parameters.

### 3.6. Taxonomic Association with Physicochemical Parameters

**Figure 6:**
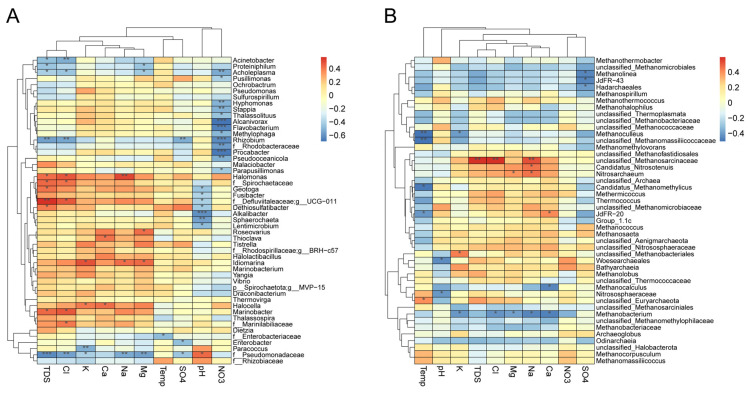
Heatmap of Spearman’s correlation between relative abundances of microbial taxa and physiochemical parameters. (**A**) taxonomic association with physicochemical parameters of the top 50 bacterial genera. (**B**) taxonomic association with physiochemical parameters of archaeal genera. Negative correlation and positive correlations are indicated by blue and red colors, respectively. * indicates 0.01 < *p* ≤ 0.05, ** indicates 0.001 < *p* ≤ 0.01, *** indicates *p* ≤ 0.001.

To determine the influence of physiochemical parameters on the relative abundances of individual microbial taxa, we applied Spearman’s correlation analysis between physiochemical parameters and the relative abundances of microbial taxa at the genus level. We found that 34 of the top 50 bacterial genera significantly correlated with at least one physiochemical parameter. Of these genera, 13 bacterial genera including *Alcanivorax*, *Flavobacterium*, *Rhizobium*, and *Procabacter* negatively correlated with NO_3_^−^ and no genus of the top 50 genera showed a positive correlation with NO_3_^−^ (Figure 6A). This result might explain why bacterial alpha diversity decreased as nitrate concentration increased. The correlation analysis also showed that seven out of the top 50 bacterial genera including *Alkalibacter* and *Sphaerochaeta* were negatively correlated with the pH value (Figure 6A). Only one genus (i.e., an unclassified genus in the family of *Pseudomonadaceae*) was positively correlated with pH. It was notable that the pH value and TDS (including cations and chloride) showed opposite effects on the relative abundances of the bacterial taxa.

Compared with bacterial taxa, the correlation between archaeal taxa and physiochemical parameters was weaker (Figure 6B). Of all archaeal genera, only 16 out of all 46 archaeal genera were significantly correlated with at least one physicochemical parameter. An unclassified genus in the family *Methanosarcinaceae* was positively correlated with TDS, chloride, and sodium, suggesting that this genus might be resistant to high saline conditions. In contrast, the genus *Methanobacterium* was less resistant to saline conditions, which decreased when the concentrations of chloride and sodium were increased. These results were consistent with low beta diversity within archaeal communities, which also suggested that archaeal communities in oil reservoirs were stable and homogeneous at a localized geographic scale.

## 4. Conclusions

In this work, we investigated the microbial communities in different oil production wells from the same oil field. We found that both bacterial and archaeal communities are dispersal limited, even at the localized geographic scale. Compared with the bacterial communities, the archaeal communities were more similar within different production wells. Further correlation analysis also showed that nitrate concentration negatively correlated with bacterial alpha diversity and pH differences positively correlated with the dissimilarity of bacterial communities. Our results suggest that the assembly of microbial communities in oil reservoirs is scale- and taxonomy-dependent. Our work provides new insights into the assembly of microbial communities in oil reservoirs.

## Figures and Tables

**Table 1:** Physicochemical parameters of the samples.

Sample ID	Type *	Depth (m)	Temperature (°C)	pH	TDS (mg/L)	Cl^−^ (mg/L)	NO_3_^−^ (mg/L)	SO_4_^2−^ (mg/L)	Na^+^ (mg/L)	K^+^ (mg/L)	Mg^2+^ (mg/L)	Ca^2+^ (mg/L)
Z1	IW	/	/	7.42	800	204	31	146	9	<1	4	5
Z3	IW	/	/	7.74	533	1069	6	128	40	<1	4	10
Z4	IW	/	/	7.80	567	594	12	75	23	<1	3	4
L68-1	PW	1773	61	6.76	15,867	7400	11	3	1800	43	60	752
L77-1	PW	1570	56	6.38	8800	5025	11	2	2684	30	68	522
L78-1	PW	1707	60	6.98	28,467	9900	22	13	2211	40	104	639
L79-1	PW	1739	60	6.30	36,000	13,920	22	64	2942	72	71	716
L79-2	PW	1711	60	6.75	30,667	9055	9	9	3569	29	70	1164
L79-3	PW	1505	54	6.84	30,167	14,195	9	2	5485	85	178	1767
L79-4	PW	1700	60	6.94	27,333	10,605	8	8	5360	58	107	1146
L83-1	PW	1519	54	6.82	33,733	17,185	9	3	8224	85	194	2206
L83-2	PW	1654	58	6.77	38,967	19,745	8	22	5653	294	80	1037
L84-1	PW	1563	55	6.60	42,567	19,855	48	31	7915	88	198	1739.5
L84-2	PW	1483	53	6.86	42,200	21,325	10	21	10,138	220	315	2748.5
L85-1	PW	1549	55	6.96	33,467	12,340	8	14	4693	70	145	1235
L87-1	PW	1700	60	6.64	37,000	15,980	237	276	10,380	146	286	2112
L87-2	PW	1742	60	6.66	50,867	24,250	179	36	9050	46	128	1149
L87-3	PW	1646	58	7.07	40,167	17,470	191	18	6970	43	109	1534
L90-1	PW	1666	58	6.73	33,433	12,950	190	54	6505	114	180	1258
L90-2	PW	1645	58	6.45	89,467	40,960	229	53	20,450	199	690	3469
L90-3	PW	1807	62	6.84	32,667	14,980	201	30	6975	105	196	1345
L1	PW	1773	62	6.48	58,967	24,650	57.	3	10,598	111	281	3436
L2	PW	1581	55	6.83	57,000	25,050	43	254	12,580	113	333	4010
L3	PW	1802	62	6.43	23,533	10,150	4	8	4544	114	118	1151
L4	PW	1566	56	6.56	48,100	16,350	6	19	6650	92	189	1969
L5	PW	1590	57	6.82	39,733	19,295	0	5	21,370	85	253	2007
L6	PW	1699	59	7.20	12,833	4997.5	10	768	6798	74	211	1817
L7	PW	1781	61	6.50	47,333	18,670	23	513	3526	84	204	444
L8	PW	1513	53	6.76	36,000	12,540	16	11	2096	33	61	575

* IW, injected water; PW, produced water.

**Table 2:** Mantel tests using Spearman’s correlation (permutations = 999) of weighted UniFrac dissimilarities between all communities sampled and each physiochemical parameter.

Factors	Mantel r Statistic	*p*-Value	Tail Type
Bacteria			
Temp	−0.0100	0.521	two-sided
pH	0.2876	0.005	two-sided
TDS	0.0351	0.364	two-sided
Cl^−^	0.0098	0.440	two-sided
NO_3_^−^	−0.0564	0.648	two-sided
SO_4_^2−^	−0.0376	0.581	two-sided
Na^+^	−0.1168	0.846	two-sided
K^+^	−0.1374	0.889	two-sided
Mg^2+^	−0.1239	0.883	two-sided
Ca^2+^	−0.1316	0.888	two-sided
Archaea			
Temp	0.0511	0.228	two-sided
pH	0.0629	0.239	two-sided
TDS	0.1313	0.133	two-sided
Cl^−^	0.0724	0.225	two-sided
NO_3_^−^	0.0974	0.179	two-sided
SO_4_^2−^	0.0621	0.287	two-sided
Na^+^	−0.0272	0.560	two-sided
K^+^	−0.0885	0.792	two-sided
Mg^2+^	−0.0269	0.569	two-sided
Ca^2+^	−0.0098	0.487	two-sided

## Data Availability

The 16S rRNA gene high throughput sequencing data were deposited in the National Microbiology Data Center (NMDC) under the accession numberNMDC10018269.

## Supplementary Materials

The following supporting information can be downloaded at: https://www.mdpi.com/article/10.3390/microorganisms11010151/s1. Figure S1: Distribution of bacterial (A) and archaeal (B) ASVs in the produced water and injected water; Figure S2: Bacterial composition of samples at the phylum level; Figure S3: Relationships between bacterial alpha diversities and physiochemical parameters; Figure S4: Relationships between archaeal alpha diversities and physiochemical parameters; Figure S5: Relationships between bacterial profiles and physiochemical parameters; Figure S6: Relationships between weighted UniFrac distances of bacterial communities and variations in physiochemical parameters; Figure S7: Relationships between archaeal profiles and physiochemical parameters; Figure S8: Relationships between weighted UniFrac distances of archaeal communities and variations in physiochemical parameters; Table S1: Hydrodynamic connection of injection and production wells used in the work.
