# Supplementary material for: pH and Nitrate Drive Bacterial Diversity in Oil Reservoirs at a Localized Geographic Scale
Source: Microorganisms. 2023 Jan 6;11(1):151. doi: 10.3390/microorganisms11010151 (PMC9865607; doi:10.3390/microorganisms11010151)
Supplement: Supplementary file 1 [file microorganisms-11-00151-s001.zip › microorganisms-2112854-supplementary.docx]

Article

pH and nitrate drive bacterial diversity in oil reservoirs at a localized geographic scale

Ying Xu^1,#^, Jianwei Wang^2,#^, Qingjie Liu^1^, Qun Zhang ^1^, Jiazhog Wu^1^, Minghui Zhou^1^, Yong Nie^2,*^, Xiao-Lei Wu^2,3,4*^

^1^ State Key Laboratory of Enhanced Oil Recovery, PetroChina Research Institute of Petroleum Exploration & Development, Beijing 100083, China

^2^ College of Engineering, Peking University, Beijing 100871, China

^3^ Institute of Ocean Research, Peking University, Beijing 100871, China

^4^ Institute of Ecology, Peking University, Beijing 100871, China

^#^ These authors contributed equally to this work

***** Correspondence: nieyong@pku.edu.cn (Y.N.), xiaolei_wu@pku.edu.cn (X.L.W)


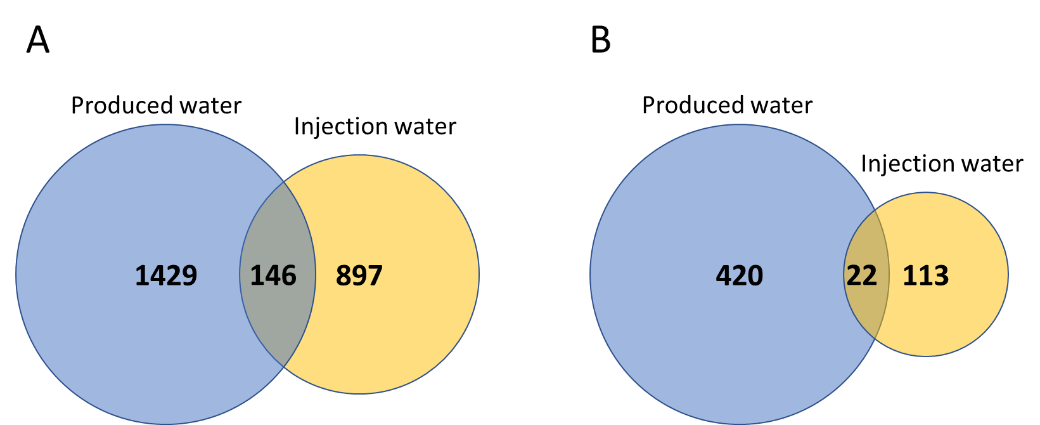


**Figure S1** Distribution of bacterial (A) and archaeal (B) ASVs in the produced water and injection water.


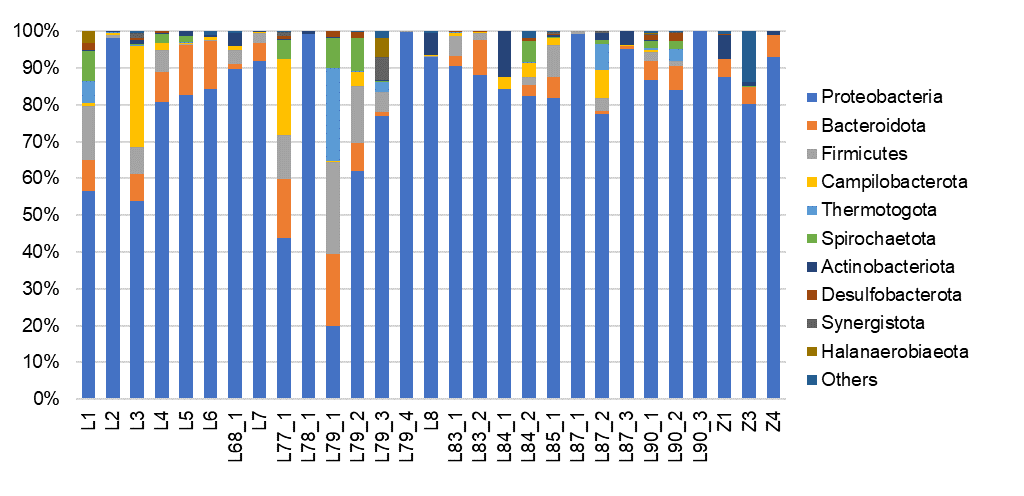


**Figure S2** Bacterial composition of samples at the phylum level.


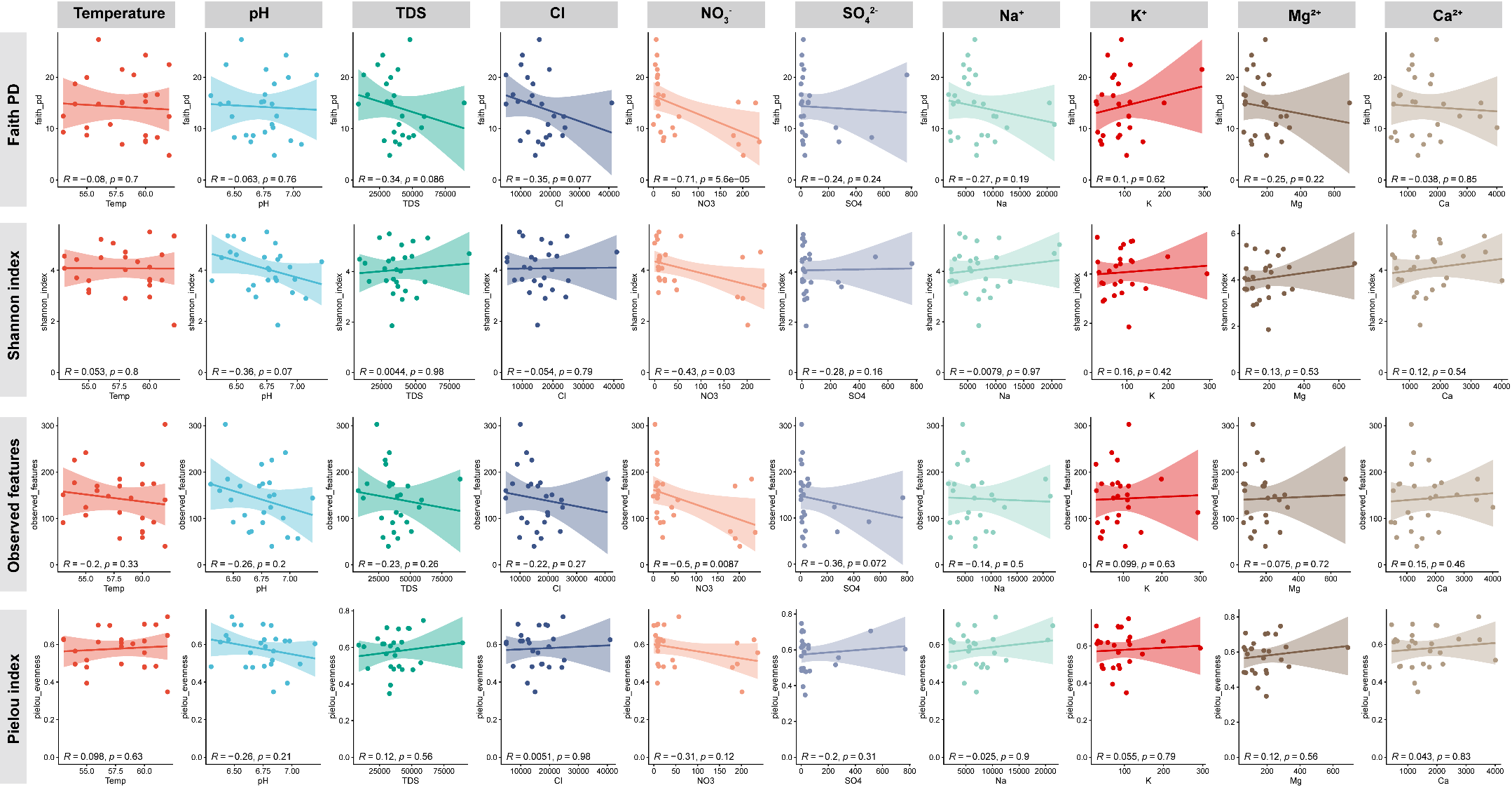


**Figure S3** Relationships between bacterial alpha-diversities and physiochemical parameters.


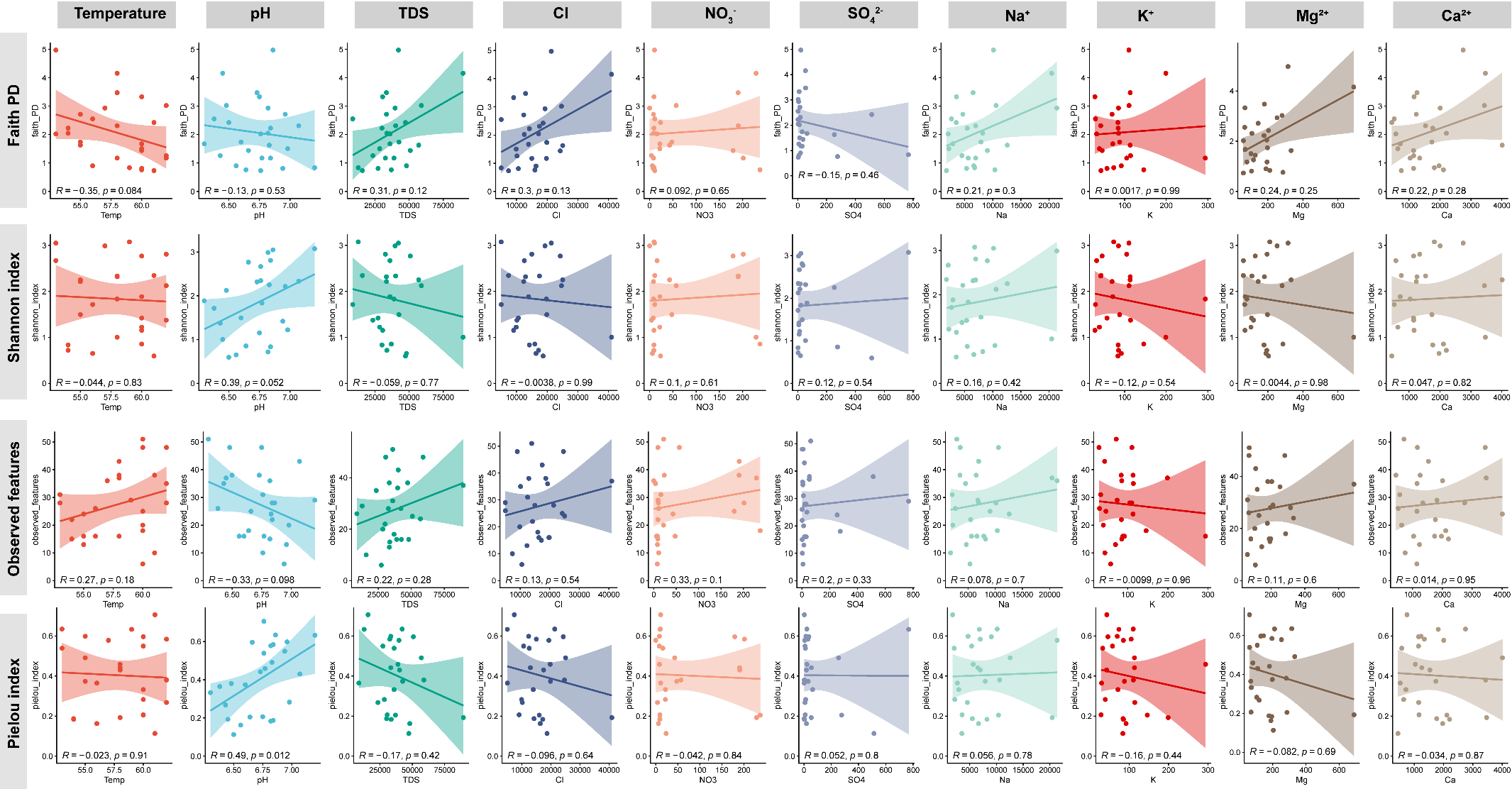


**Figure S4** Relationships between archaeal alpha-diversities and physiochemical parameters.

**Figure S5** Relationship between bacterial profiles and physiochemical parameters.

**Figure S6** Relationships between weighted UniFrac distances of bacterial communities and variations in physiochemical parameters.

**Figure S7** Relationship between archaeal profiles and physiochemical parameters.

**Figure S8** Relationships between weighted UniFrac distances of archaeal communities and variations in physiochemical parameters.

**Table S1** Hydrodynamic connection of injection and production wells used in the work

| **Injection well** | **Production well** |
| --- | --- |
| Z1 | L77-1, L79-2, L79-3, L79-4, L85-1, L2, L3, L4 |
| Z3 | L68-1, L78-1, L79-1, L83-1, L83-2, L1, L5, L6, L7, L8 |
| Z4 | L84-1, L84-2, L87-1, L87-2, L87-3, L90-1, L90-2, L90-3 |
